# Acute pain after total hip and knee arthroplasty does not affect chronic pain during the first postoperative year: observational cohort study of 389 patients

**DOI:** 10.1007/s00296-022-05094-4

**Published:** 2022-02-26

**Authors:** D. A. J. M. Latijnhouwers, C. H. Martini, R. G. H. H. Nelissen, S. H. M. Verdegaal, T. P. M. Vliet Vlieland, M. G. J. Gademan, the Longitudinal Leiden Orthopaedics Outcomes of Osteoarthritis Study (LOAS) Group, H. M. J. van der Linden, B. L. Kaptein, P. J. Damen, H. H. Kaptijn, S. B. W. Vehmeijer, W. C. M. Marijnissen, R. Onstenk

**Affiliations:** 1 Department of Orthopedics, Leiden University Medical Center, Leiden, The Netherlands; 2 Department of Anesthesiology, Leiden University Medical Center, Leiden, The Netherlands; 3 Department of Orthopedics, Alrijne Hospital, Leiderdorp, The Netherlands; 4 Department of Orthopedics, Rehabilitation and Physical Therapy, Leiden University Medical Center, Leiden, The Netherlands; 5 Department of Clinical Epidemiology, Leiden University Medical Center, Leiden, The Netherlands

**Keywords:** Hip, Knee, Arthroplasty, Acute, Chronic, Pain

## Abstract

Chronic pain is frequently reported after total hip and knee arthroplasties (THA/TKA) in osteoarthritis (OA) patients. We investigated if severity of acute postoperative pain following THA/TKA in OA patients was associated with pain during the first postoperative year. From an observational study, OA patients scheduled for primary THA/TKA (June 2012–December 2017) were included from two hospitals in the Netherlands. Acute postoperative pain scores were collected within 72 h postoperatively and categorized as no/mild (NRS ≤ 4) or moderate/severe (NRS > 4). Pain was assessed preoperatively, 3, 6 and 12 months postoperatively using the HOOS/KOOS subscale pain. With Multilevel Mixed-effects-analyses, we estimated associations between acute and chronic pain until one year postoperative, adjusted for confounders and including an interaction term (Time*Acute pain). 193 THA and 196 TKA patients were included, 29% of THA and 51% of TKA patients reported moderate/severe pain acutely after surgery. In the THA group, the difference in pain at 3 months between the no/mild and moderate/severe groups, was approximately six points, in favor of the no/mild group (95% CI [−12.4 to 0.9]) this difference became smaller over time. In the TKA group we found similar differences, with approximately four points (95% CI [−9.6 to 1.3]) difference between the no/mild and moderate/severe group at 6 months, this difference attenuated at 12 months. No association between severity of acute postoperative pain and pain during the first postoperative year was found. These findings suggest that measures to limit acute postoperative pain will likely not impact development of chronic pain.

**Supplementary Information:**

The online version contains supplementary material available at 10.1007/s00296-022-05094-4.

## Introduction

Approximately 20% of the general adult population suffers from OA of the knee, hip or hand, with increasing numbers due to age and obesity, resulting in increasing numbers of patients in pain due to OA [1]. Pain is a pervasive symptom in osteoarthritis (OA) patients, more often occurring than functional limitations [1], and the main reason that patients seek medical attention [2]. A previous study including patients with knee OA, showed that 41% of patients experience mild pain, 40% experienced moderate pain and 20% experienced severe pain [3]. Additionally, Zolio et al. [4] described the prevalence of neuropathic-like pain and/or pain sensitization in patients with knee and hip OA, using self-administered questionnaires, and showed that the prevalence of these types of pain ranged between 20 and 40% in knee OA and 9–29% in hip OA patients. Nevertheless, it is difficult to provide a pain prevalence number in these patients, given the variation in pain experienced by patients, as well as pain scores obtained and heterogeneity of the studied populations [5].

Apart from the fact that patients often suffer from pain prior to surgery, chronic pain after total hip and knee arthroplasty (THA/TKA) is an often reported unfavorable outcome [6, 7], with chronic pain defined as recurring or being present for more than 3 months [8]. The presence of chronic postoperative pain after arthroplasty surgery could have substantial unfavorable effects, including delayed postoperative rehabilitation [9], a negative impact on quality of life [10], decreased postoperative satisfaction [11] and increased the risk of revision surgery and additional healthcare costs [12, 13]. Therefore, new treatments targeting the prevention of the development of chronic postoperative pain after total joint arthroplasties are of utmost importance.

In surgical patients, (intensity of) acute postoperative pain has been postulated as a risk factor for chronic postoperative pain, although results are somewhat heterogeneous. [14]. Nevertheless, the effect of acute postoperative pain may be different in hip and knee arthroplasty patients compared to other surgical fields, as the majority of OA patients experienced pain for years before arthroplasty surgery [15]. Hence, one of the main indications for joint arthroplasty is chronic preoperative pain [16]. The few studies on the effect of acute postoperative pain in THA and TKA patients did not provide unequivocal evidence of the effect of acute postoperative pain on chronic postoperative pain in patients with OA [17–21]. This may be due to several reasons: the use of a cross-sectional study design, recall bias in the intensity of acute postoperative pain (reported months after surgery) or end-points analysis at 6 months, while THA and TKA patients are known to show improvements later than 6 months after surgery [22, 23]. Additional research to gain further insight into the association between acute and chronic postoperative pain in THA and TKA patients is therefore needed. If the intensity of acute postoperative pain is associated with chronic postoperative pain, prevention of severe acute postoperative pain could be a treatment target to improve chronic postoperative pain.

Consequently, we performed a longitudinal, multi-center study to investigate if severity of acute postoperative pain, following THA or TKA in OA patients, is associated with chronic pain during the first postoperative year.

## Materials and methods

### Patients

This study was part of the ongoing cohort Longitudinal Leiden Orthopaedics and Outcomes of Osteoarthritis Study (LOAS) (Trial ID NTR3348) [24]. In short, ethical approval for the LOAS was obtained prior to patient recruitment from the Medical Ethics Committee of Leiden University Medical Center (LUMC; P12.047, date: 27th of March, 2012). Patients with OA and scheduled for a primary THA or TKA at two hospitals (i.e., Leiden University Medical Center (LUMC) or Alrijne Hospital) between June 2012 and December 2017 were included in the LOAS, after informed consent was obtained, which is in compliance with the Helsinki Declaration. For the present study, patients without an acute postoperative pain score or without any follow-up measurements were excluded.

### Pain assessments

Acute pain scores were assessed by a nurse and reported in the medical file, every three hours within 72 h after surgery (during hospitalization), using the Numeric Rating Scale (NRS) at rest. This was part of standard care in both hospitals. The NRS provides a number between 0 and 10, with 0 meaning no pain and 10 meaning the worst pain possible [25]. An average acute pain score was calculated based on the two highest pain scores of all available pain scores within 72 h after surgery and afterwards categorized into ‘no/mild’ if NRS ≤ 4 and ‘moderate/severe’ if NRS > 4 [26, 27]. If patients reported pain score > 4 points, additional analgesia were provided, as an NRS > 4 is seen as postoperative pain in need of intervention in the hospitals included in this study. Additionally, NRS > 4 served as a contraindication for discharge from recovery. Even more, a pain score of NRS 4 in postoperative patients is used as an upper limit (i.e. benchmark) in quality measure assessments.

### Analgesic treatment

Patients for THA received spinal, combined spinal-epidural or general anesthesia during surgery. Postoperative pain relief was achieved by a combination of paracetamol, NSAID and morphine subcutaneously. Patients were discharged with oral oxycodone. TKA was performed under spinal, combined spinal-epidural or general with epidural anesthesia. Postoperatively, patients received a combination of paracetamol, NSAID and a patient controlled epidural anesthesia (PCEA) pump or an intravenous patient controlled anesthesia (PCA) pump with either morphine or fentanyl in case of spinal anesthesia. After epidural anesthesia was terminated, morphine was subcutaneously administered. Patients were discharged with oral oxycodone. Using questionnaires, patients were asked to report the use of acetaminophen or non-steroidal anti-inflammatory drugs (NSAIDs) in the past six months before surgery, because of hip or knee complaints ((almost) daily, few days per week, few days per month), and indicate the persistence of hip or knee joint symptoms (< 1 year, 1–5 years, 5–10 years and > 10 years). Additionally, duration of surgery (minutes) and hospitalization (days) were collected from the medical records as possible proxies for complications during and after surgery [28].

Prior to surgery, and at 3 (if THA), 6, and 12 months after surgery patients received questionnaires to obtain pain scores using validated Dutch versions of the Hip disability of Knee injury and Outcomes of Osteoarthritis Score (HOOS/KOOS) [29]. These questionnaires contain 40 and 42 items, respectively, categorized into five subscales. We used the HOOS/KOOS pain subscale, with scores ranging from 0 to 100, with 0 indicating ‘extreme pain’ and 100 indicating ‘no pain’.

### Secondary outcome measurements

The following sociodemographic characteristics were collected: age, sex, Body Mass Index (BMI (kg/m^2^)), type of anesthesia (i.e., local, general, or combination), living arrangement (household composition; i.e., living alone (yes/no)) and working status (i.e., employed or unemployed). To indicate the mental health status of the patient, the subscale Mental Component Summary of the Short-Form-12 (MCS-12) (ranging from 0 to 100, with higher scores representing better health) was used [30]. Information on existing comorbidities was collected with the comorbidity questionnaire of the Dutch Central Bureau of Statistics (CBS) [31], asking for the presence or absence of comorbidities in the previous year (yes/no). Comorbidities were classified into three domains: musculoskeletal comorbidities (severe elbow, wrist, hand or back pain and other rheumatic diseases), non-musculoskeletal comorbidities (chronic lung, cardiac, or coronary disease; arteriosclerosis; hypertension; stroke; severe bowel disorder; diabetes mellitus; migraine; psoriasis; chronic eczema; cancer; incontinence; hearing or vision impairments; and dizziness in combination with falling) and comorbidities that could cause chronic pain syndromes (diabetes mellitus; migraine; back pain, other rheumatic diseases).

### Sample size calculation

We anticipated a correlation of 0.2 between severity of acute postoperative pain and pain within the first year after surgery. The sample size calculation with alpha equal to 0.05 and 80% power, resulted in a required sample size of 193 patients for the THA and TKA group.

**Fig. 1:**
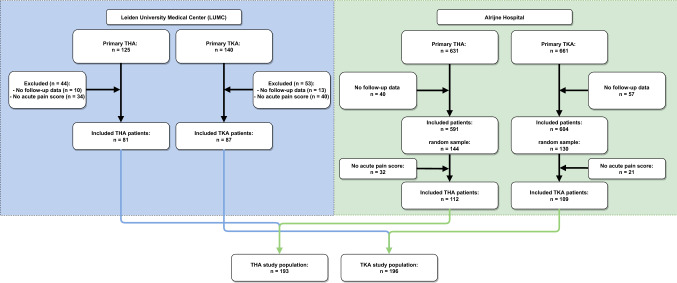
Flowchart of included total hip and knee arthroplasty patients. *THA* Total Hip Arthroplasty, *TKA* Total Knee Arthroplasty

First, we assessed all 265 patients included in the LUMC between July 2012 and December 2017 (THA: *n* = 125; TKA: *n* = 140). Of these patients, 23 had no follow-up data, and 74 had no acute pain scores available, resulting in 168 LUMC patients (THA: *n* = 81; TKA: *n* = 87) to be included (Fig. 1). Then, we included a random sample from the Alrijne Hospital THA and TKA population to reach a minimum of 193 THA patients and 196 TKA patients (Fig. 1).

### Statistical analyses

All analyses were performed while stratified by joint (hip or knee). Patients were grouped according to their acute postoperative score (i.e., no/mild NRS ≤ 4, moderate/severe NRS > 4). To compare the characteristics of patients with no/mild and moderate/severe acute postoperative pain, we performed Mann–Whitney *U* tests (after check for normality, non-parametric tests were performed for continuous variables) and Chi-Square tests (for proportions). Baseline characteristics of included and excluded patients were compared to assess for selection bias. Although not statistically tested to avoid multiple testing, no clinical important differences were found between included and excluded patients (Supplementary Table 1).

We used multilevel mixed-effects-analyses to assess whether severity of acute postoperative pain was associated with pain during the first postoperative year, because it takes the between-patient correlation into account. The models included a group variable based on acute postoperative pain (i.e., no/mild or moderate/severe) and a time variable (i.e., 3 months, 6 months, 12 months). The models included subject-specific intercepts and a random effect to account for the correlation between repeated measures over time in the same patient, while correcting for originating hospital as patient population could vary between both hospitals. By including an interaction term between acute postoperative pain and timing of postoperative pain measurement (evaluated at 3 (if THA), 6 and 12 months postoperatively), we were able to assess whether acute postoperative pain affected postoperative pain during the first year after surgery. Further, we corrected for several confounders: age, sex, BMI, preoperative HOOS/KOOS pain score, preoperative MCS-12 score, surgery duration, hospitalization duration/length and type of anesthesia [18, 32]. Missing values in preoperative pain (1.6% in THA-group and 5.6% in TKA-group) and MCS-12 (2.6% in THA-group and 3.6% in TKA-group) (assumed to be missing at random (MAR)) were imputed using the Multivariate Imputation by Chained Equations (MICE) algorithm in R, based on variable available in the final model. Crude versus imputed models did not show differences in associations (data not shown). Sensitivity analyses were performed while including a continuous value for acute pain instead of acute pain based on the cut off value, to test the robustness of our exposure assessment. The effect estimates were depicted as coefficients with 95% Confidence Intervals (95%-CI). All analyses were performed using R (Version R 3.6.1).

## Results

A total of 193 THA (57% female, mean age 67 (SD 10.4)) and 196 TKA patients (63% female, mean age 66 (SD 8.4)) were included in the present study (Fig. 1, Table 1). The median number of acute postoperative pain measurements per patient was 5 [range 1–13] in THA and 6 [range 1–17] in TKA patients. In 15 THA and 14 TKA patients, only one NRS measurement was recorded. Overall, the mean acute postoperative pain scores were 4 (SD 1.7) in THA patients and 5 (SD 2.0) in TKA patients. Almost a third of the THA patients reported moderate/severe pain (NRS > 4 = 29%), compared to half of the TKA patients (NRS > 4 = 51%). THA patients reporting moderate/severe pain scored worse on preoperative pain and MCS-12, had increased duration of surgery and hospitalization period, and more often received a cemented arthroplasty compared to the no/mild acute pain group (Table 1). TKA patients reporting moderate/severe pain scored worse on preoperative pain, used more acetaminophen 6 months prior to surgery, had lower MCS scores, and had a longer duration of surgery and hospitalization period compared to the no/mild acute pain group (Table 1).

**Table 1:** Comparison of preoperative patient characteristics and peri and postoperative treatment information: stratified for mild (NRS < 5) and moderate/severe (NRS ≥ 5) acute pain and joint

	THA				TKA			
	Total population (*n* = 193)	Mild (*n* = 137)	Moderate/Severe (*n* = 56)	*p* value^a^	Total population (*n* = 196)	Mild (*n* = 97)	Moderate/severe (*n* = 99)	*p* value^a^
**Sex,** Female; *n* (%)	109 (57)	76 (56)	33 (59)	0.66	124 (63)	58 (60)	33 (67)	0.32
**Age, median (IQR)** (years)	66.0 (12)	66.5 (13)	66.0 (12)	0.97	66.0 (11)	65.0 (10)	66.0 (12)	0.56
**BMI, median (IQR)**	26.3 (6)	26.3 (6)	26.7 (6)	0.64	28.4 (6)	28.4 (6)	28.3 (7)	0.83
**HOOS/KOOS, median (IQR) **Preoperative pain (0–100)	35.0 (28)	37.5 (27)	27.5 (27)	0.003	36.1 (19)	40.4 (19)	33.3 (19)	0.002
**Acetaminophen,** *n* (yes (%))	119 (62)	81 (59)	38 (70)	0.35	143 (73)	60 (62)	39 (80)	0.004
**NSAIDs,** *n* (yes (%))	76 (39)	55 (40)	21 (38)	0.60	87 (44)	35 (36)	21 (43)	0.08
**Duration of complaints**, *n* (%) (years)								
< 1	28 (15)	17 (12)	17 (12)	0.51	10 (5)	3 (3)	7 (7)	0. 98
1–5	106 (55)	76 (56)	76 (53)		87 (44)	45 (46)	42 (42)	
5–10	28 (15)	19 (14)	19 (14)		39 (20)	18 (19)	21 (21)	
> 10	16 (8)	13 (10)	13 (10)		44 (22)	21 (22)	23 (23)	
**MCS-12, median (IQR)** (0–100)	54.2 (14)	54.8 (13)	52.6 (16)	0.04	53.7 (10)	56.8 (10)	52.5 (9)	0.03
**Work,** *n* (yes (%))	56 (29)	41 (30)	15 (27)	0.69	63 (32)	32 (33)	31 (31)	0.76
**Comorbidities**, *n* (%)								
Non-musculoskeletal	47 (24)	33 (24)	10 (18)	0.62	51 (26	26 (27)	25 (25)	0.90
Musculoskeletal	1 (1)	1 (1)	14 (25)		–	–	–	
Chronic pain syndrome related	38 (20)	28 (20)	10 (18)		32 (16)	14 (14)	18 (18)	
All	66 (34)	43 (31)	23 (41)		69 (35)	31 (32)	38 (38)	
None	21 (11)	17 (12)	4 (7)		17 (9)	8 (8)	9 (9)	
**Living arrangement**, *n* (%)								
Alone	49 (25)	31 (23)	18 (32)	0.77	39 (20)	13 (13)	26 (26)	0.31
**Type of anesthesia**, *n* (%)								
General	37 (19)	21 (15)	16 (29)	0.35	25 (13)	9 (9)	16 (16)	0.06
Local	31 (16)	26 (19)	5 (9)		86 (44)	40 (41)	46 (47)	
Combination	125 (65)	90 (66)	35 (63)		85 (43)	48 (50)	37 (37)	
**Fixation**, *n* (%)								
Cementless	133 (69)	103 (75)	30 (54)	0.02	9 (5)	6 (6)	3 (3)	0.21
Hybrid	8 (4)	5 (4)	3 (5)		2 (1)	2 (2)	–	
Cemented	51 (26)	29 (21)	22 (39)		180 (92)	88 (91)	92 (93)	
Missing	1 (1)	–	1 (2)		5 (3)	1 (1)	4 (4)	
**Duration surgery, median (IQR)** (min)	72 (50)	70 (51)	88 (40)	0.004	77 (34)	68 (30)	82 (38)	< 0.001
**Hospitalization, median (IQR)** (days)	3 (2)	2 (2)	3 (4)	< 0.001	3 (2)	2 (2)	3 (3)	< 0.001
**Acute pain NRS, median (IQR)** (0–10)	4 (2)				5 (3)			

All continuous variables are depicted as median (Interquartile Range (IQR))

*NRS* Numeric Rating Scale, *THA* Total Hip Arthroplasty, *TKA* Total Knee Arthroplasty, *n* number of patients, *BMI* Body Mass Index, *HOOS* The Hip disability and Osteoarthritis Outcome Score, *KOOS* The Knee injury and Osteoarthritis Outcome Score, *NSAID* Non-steroidal Anti-inflammatory Drugs, *MCS-12* Mental Component Summary of the Short-Form 12

^a^Comparison of patients with mild and moderate/severe acute pain by means of Mann–Whitney *U* tests (for continuous variables) or Chi Square test (for categorical variables)

### Association acute postoperative pain

**Fig. 2:**
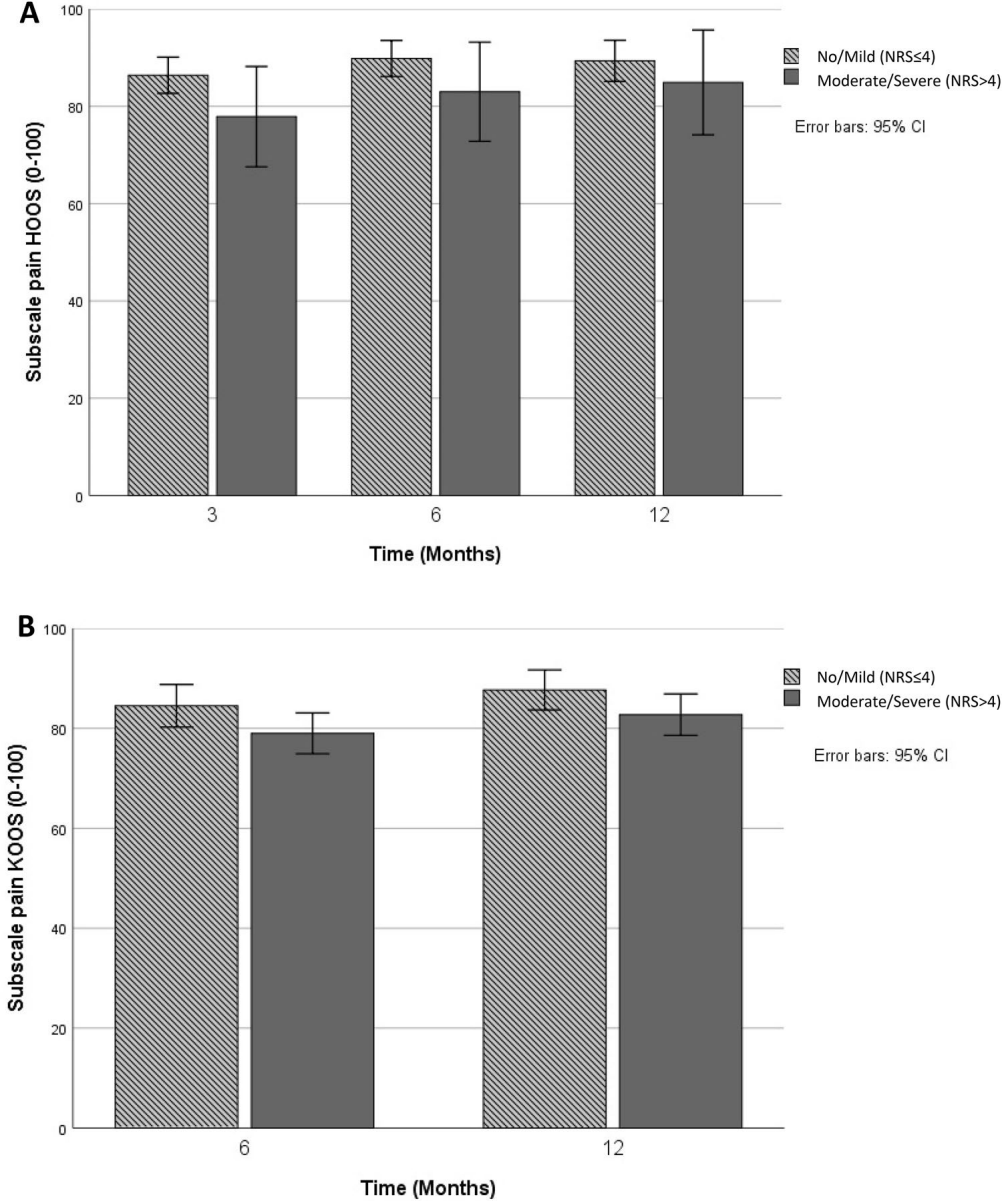
**A** HOOS pain scores at 3, 6 and 12 months postoperatively in Total Hip Arthroplasty patients reporting mild or severe acute pain (0 = extreme pain, 100 = no pain) (*n* = 83). *HOOS* Hip disability and Osteoarthritis Outcome Score. **B** KOOS pain scores at 6 and 12 months postoperatively in Total Knee Arthroplasty patients reporting mild or severe acute pain (0 = extreme pain, 100 = no pain) (*n* = 148). *KOOS* Knee injury and Osteoarthritis Outcome Score

To evaluate if acute pain severity was associated with pain during the first postoperative year, an interaction term (Acute pain*Time of measurement) was added to the models. In the THA group, the difference between the no/mild and moderate/severe groups, was approximately 6 points (95%-CI [−12.4 to 0.9], on a 0–100 scale) at 3 months in favor of the no/mild group, and the difference became smaller over time. In the TKA group we found similar differences, with approximately 4 points (95%-CI [−9.6 to 1.3], on a 0–100 scale) difference between the no/mild and moderate/severe group at 6 months. The differences attenuated at 12 months. None of the coefficients showed clinically or statistically significant differences between no/mild and moderate/severe acute postoperative pain and postoperative pain during the first postoperative year (Table 2). There were also no postoperative differences present between the no/mild and moderate/severe groups when compared in patients with complete follow-up (Fig. 2A and B).

**Table 2:** Estimated effects of the association between severity of acute postoperative pain and postoperative pain over time in total hip and knee arthroplasty patients

	THA Coefficient [95% CI]^a^	TKA Coefficient [95% CI]^a^
Adjusted model^b^		
Moderate/severe acute pain	− 5.7 [− 12.4 to 0.9]	− 4.1 [− 9.6 to 1.3]
6 months	5.5 [0.5 to 10.5]	
Acute pain*6 months	1.5 [− 4.5 to 7.5]	
12 months	− 1.7 [− 6.8 to 3.3]	2.5 [− 2.2 to 7.2]
Acute pain*12 months	4.8 [− 1.2 to 10.8]	1.2 [− 3.7 to 6.1]

Mixed model including interaction term hospital*time and acute pain*time; mild acute pain as reference category

*THA* Total Hip Arthroplasty, *TKA* Total Knee Arthroplasty

^a^95% CI = 95% Confidence Interval

^b^Adjusted for Sex, Age, BMI, Preoperative pain, MCS-12, Duration of surgery and Hospitalization, Type of Anesthesia

### Sensitivity analyses

In supplementary Table 2 the findings from the sensitivity analyses are shown. Including acute pain as a continuous measure in our model instead of including acute pain as category (NRS ≤ 4 or NRS > 4) yielded similar results.

## Discussion

In this study we investigated if severity of acute postoperative pain, following THA or TKA in OA patients, was associated with pain during the first postoperative year. Almost one-third of THA, and more than half of TKA patients reported moderate/severe acute postoperative pain. Nevertheless, our findings indicated that severity of acute postoperative pain after THA or TKA was not associated with postoperative pain during the first postoperative year in this population.

Our findings are consistent with previous studies that were unable to show an association between acute and chronic postoperative pain in THA and TKA patients [17, 20, 21]. Similar to our study these studies collected acute postoperative pain scores shortly after surgery (within the first two postoperative days). In contrast to our study, both studies were smaller and assessed postoperative pain only up to 6 months after surgery, which is similar to the duration of follow-up in the study of Buvanendran et al. [18], who did find an effect of acute pain on postoperative pain. In comparison with Buvanendran et al. [18], we had a longer follow-up, included postoperative pain as a continuous measure, and applied less strict inclusion criteria. The study of Nikolajsen et al. [19] also found an effect of acute pain on postoperative pain, but patients had to recall their acute postoperative pain 12–18 months postoperatively, thereby possibly introducing recall bias. Our study collected acute postoperative pain from the medical files, reported during their hospitalization within 72 h after surgery.

While our study is in concordance with some studies that assessed the effect of acute pain in OA patients after THA and TKA, our findings are inconsistent with other surgical fields that consider intensity of acute postoperative pain as a risk factor for the development of chronic pain [33]. A possible explanation could be that the type of injuries or diseases in these surgical fields have a more acute onset compared to patients suffering from OA, as patients with OA often suffer from (chronic) pain prior to surgery. This pain is the main reason that OA patients seek medical attention [2]. Previous research showed that OA pain is the result of the involvement of both peripheral and central processes [2]. Nevertheless, the mechanism of pain is complex and not fully understood in OA patients, and neither is the development of chronic pain in patients after THA and TKA.

It is known that chronic pain is associated with changes in the peripheral and central nervous system in response to acute injury, such as surgery or trauma. This could result in hypersensitivity of the nervous system and if persistent, lead to central sensitization [34]. Several studies showed that central sensitization is related to the most consistent risk factors for the development of chronic postoperative pain in OA patients: preoperative and subacute (within 4–12 weeks after surgery) postoperative pain [32, 35–37]. Additionally, subacute pain triggers peripheral sensitization, which could result in central sensitization [38]. According to previous studies, the development of chronic postoperative pain may be prevented by preventing the development of central sensitization preoperatively or identify patients already suffering from central sensitization before surgery, as patients with central sensitization often experience less benefits after joint arthroplasties [33, 39].

As chronic pain could be the result of either chronic preoperative pain or (sub)acute postoperative pain, it is difficult to differentiate whether chronic postoperative pain is the result of (sub)acute postoperative pain from the surgery or ongoing preoperative pain. Future cohort studies including more extensive measurements for study purposes on preoperative and (sub)acute postoperative pain, preoperative data on endogenous pain modulation and quantitative sensory testing including central sensitization [40], should be carried out to contribute to the knowledge on modifiable factors of chronic postoperative pain after THA and TKA. Understanding the multifactorial components of OA pain and identifying possible causes of pain in these patients could result in more appropriate and effective treatments to help decrease the prevalence of chronic pain.

Pain experience in OA has a multidimensional nature, which causes the underlying etiology of OA-pain also to be multifactorial. Several modifiable risk factors have been reported that could reduce pain before surgery: losing weight, healthier dietary choices, levels of physical activity, use of assistive devices (i.e. insoles), decrease the number of medical comorbidities, pain catastrophizing and coping, and psychological factors, such as anxiety, distress and depression might also affect postoperative pain [5, 32, 41, 42]. Sorel et al. [42] showed that perioperative interventions targeting psychological distress resulted in improved pain scores or decreased opioid or other types of pain medication prescriptions after TKA. Lastly, decreasing preoperative pain could result in better postoperative outcomes regarding pain in this population, as preoperative pain is mentioned as one of the main risk factors of pain postoperatively.

This study has several strengths and limitations: We imputed missing values in confounders, therefore refraining from exclusion of patients with missing values. After comparing the current population with the excluded patients we found no clinically relevant differences (Supplementary Table 1). Therefore we expect that no selection bias has occurred. A possible limitation could be the method of acute postoperative pain collection from medical records, which was part of standard care. Hence, acute pain was not collected with the intention to use for study purposes, and were therefore not regularly recorded by the same nurse, which could have affected the consistency. Furthermore, there is no consensus on cut-off points for NRS pain, which might affect generalizability [27, 43–46]. However, we additionally performed a sensitivity analysis while including acute pain as a continuous exposure, which yielded similar results. Nor did this study collected subacute pain (lasting 4–12 weeks post operatively) after surgery, to measure the effect on chronic pain. Subacute pain has been identified as risk factor for chronic pain after orthopedic surgeries, especially TKA [35].We were unable to include specific information on analgesic treatment during hospitalization. However, we aimed to investigate the effect of perceived acute pain on chronic pain, making the underlying analgesic treatment less important. Additionally, we did not exclude or include patients based on their anti-inflammatory drug therapy before surgery. Lastly, some relevant patient characteristics were not available, such as the amount of pain catastrophizing, pain elsewhere in the body and presence of central sensitization in patients.

We found that a substantial group of THA and TKA patients reported moderate/severe acute postoperative pain, but no association between severity of acute postoperative pain and chronic pain during the first postoperative year was found. Although it is important to limit the presence of acute postoperative pain as much as possible, acute postoperative pain does not seem to be associated with postoperative pain. Therefore, efforts to reduce the presence of chronic pain should be focused elsewhere, such as reduction of preoperative pain, or psychological well-being of the patient.

## Supplementary Information

Below is the link to the electronic supplementary material.

Supplementary file1 (DOCX 16 KB)

Supplementary file2 (DOCX 13 KB)

## Data Availability

The data sets used and/or analyzed during the current study are available from the corresponding author on reasonable request.

## Code availability

The code used to analyze the current study are available from the corresponding author on reasonable request.
