# Supplementary material for: Acute pain after total hip and knee arthroplasty does not affect chronic pain during the first postoperative year: observational cohort study of 389 patients
Source: Rheumatol Int. 2022 Feb 26;42(4):689–98. doi: 10.1007/s00296-022-05094-4 (PMC8940785; doi:10.1007/s00296-022-05094-4)
Supplement: Supplementary file 1 — Supplementary file1 (DOCX 16 KB) [file 296_2022_5094_MOESM1_ESM.docx]

| **Supplementary table 1: Comparison of study population and patients not included: stratified by joint^a^** | | | | |
| --- | --- | --- | --- | --- |
|  | **THA** | | **TKA** | |
|  | Study population  (n = 193) | Not included  (n = 563) | Study population  (n = 196) | Not included  (n = 605) |
| **Sex,** Female; n (%) | 109 (57) | 335 (60) | 124 (63) | 379 (63) |
| **Age,** (years) **median (IQR)** | 66.0 (12) | 68.0 (13) | 66.0 (11) | 66.5 (12) |
| **BMI, median (IQR)** | 26.3 (6) | 26.6 (5) | 28.4 (6) | 28.4 (6) |
| **HOOS/KOOS,** Preoperative pain (0-100) **median (IQR)** | 35.0 (28) | 37.5 (20) | 36.1 (19) | 38.9 (23) |
| **Acetaminophen,** n (yes (%)) | 119 (62) | 363 (70) | 143 (73) | 397 (71) |
| **NSAIDs,** n (yes (%)) | 76 (39) | 232 (45) | 87 (44) | 255 (46) |
| **MCS-12,** (0-100) **median (IQR)** | 54.2 (14) | 56.3 (12) | 56.8 (10) | 56.6 (11) |
| **Work,** n (yes (%)) | 56 (29) | 151 (27) | 63 (32) | 194 (33) |
| **Supplementary table 1 Legend:**  a: All continuous variables are depicted as median (Interquartile Range (IQR))  THA = Total Hip Arthroplasty  TKA = Total Knee Arthroplasty  n = number of patients  BMI = Body Mass Index  HOOS = The Hip disability and Osteoarthritis Outcome Score  KOOS = The Knee injury and Osteoarthritis Outcome Score  NSAID = Non-steroidal Anti-inflammatory Drugs  MCS-12 = Mental Component Summary of the Short-Form-12 | | | | |
