# Supplementary material for: Acute pain after total hip and knee arthroplasty does not affect chronic pain during the first postoperative year: observational cohort study of 389 patients
Source: Rheumatol Int. 2022 Feb 26;42(4):689–98. doi: 10.1007/s00296-022-05094-4 (PMC8940785; doi:10.1007/s00296-022-05094-4)
Supplement: Supplementary file 2 — Supplementary file2 (DOCX 13 KB) [file 296_2022_5094_MOESM2_ESM.docx]

| **Supplementary Table 2: Estimated effects of the association between acute postoperative pain and postoperative pain over time in total hip and knee arthroplasty patients^a^** | | |
| --- | --- | --- |
|  | THA  Coefficient [95% CI]**^b^** | TKA  Coefficient [95% CI]**^b^** |
| **Adjusted Model^c^** | | |
| Acute pain  6 months  Acute pain*6 months  12 months  Acute pain*12 months | -1.2 [-3.0 – 0.7]  2.7 [-5.2 – 10.6]  0.9 [-0.8 – 2.5]  -5.4 [-13.5 – 2.7]  1.4 [-0.3 – 3.1] | -1.5 [-3.9 – 5.8]  -0.7 [-8.0 – 6.7]  0.8 [-0.5 – 2.0] |
|  |  |  |

**Table 2 legend:**

a: Mixed model including interaction term hospital*time and acute pain*time;

b: 95% CI=95% Confidence Interval

c: Adjusted for Sex, Age, BMI, Preoperative pain, MCS-12, Duration of surgery

and Hospitalization, Type of Anesthesia.

THA = Total Hip Arthroplasty

TKA = Total Knee Arthroplasty
